# A cross-species analysis of neuroanatomical covariance sex differences in humans and mice

**DOI:** 10.1186/s13293-025-00728-1

**Published:** 2025-07-01

**Authors:** Linh Pham, Elisa Guma, Jacob Ellegood, Jason P. Lerch, Armin Raznahan

**Affiliations:** 1 Section on Developmental Neurogenomics, Human Genetics Branch, National Institute of Mental Health, Bethesda, MD 20892 USA; 2 Mouse Imaging Centre, Toronto, ON M5T 3H7 Canada; 3 Bloorview Research Institute, Holland Bloorview Kids Rehabilitation Hospital, Toronto, ON M4G 1R8 Canada; 4 The Hospital for Sick Children, Toronto, ON M5G 1E8 Canada; 5 Department of Medical Biophysics, University of Toronto, Toronto, ON M5G 1L7 Canada; 6 Wellcome Centre for Integrative Neuroimaging, University of Oxford, Oxford, OX3 9DU UK; 7 South Texas Medical Scientist Training Program, University of Texas Health Science Center San Antonio, San Antonio, TX 78229 USA; 8 Harvard Medical School, Boston, MA 02115 USA; 9 Department of Pediatrics, Lurie Center for Autism, Massachusetts General Hospital, Lexington, MA 02421 USA

**Keywords:** Structural covariance, Anatomical correlations, Phenotypic covariation, Sex differences, Mouse, Human, Neuroimaging

## Abstract

**Background:**

Structural covariance within the brain is thought to reflect inter-regional sharing of developmental influences. This hypothesis has proved difficult to test but can be informatively probed by the study of sex differences. Here, we use neuroimaging in humans and mice to study sex-differences in anatomical covariance– asking (1) are there sex differences in structural covariance and (2) do regions that share the same developmental influences, as exhibited by shared sex differences in volume, also show shared sex differences in volume covariance. This study design illuminates both the biology of sex-differences and theoretical models for anatomical covariance– benefitting from tests of inter-species convergence.

**Methods:**

Brain volume correlations for males and females across 255 regions in mice (*n* = 423) and 378 regions in humans (*n* = 436) were calculated using volumetric measures obtained from structural MRI. Mean correlations for each sex were compared within species to determine whether covariance sex differences exist. Specific covariances with strong sex differences in each species were identified via permutation tests for statistical significance. Brain maps of regional average structural covariance sex-bias were generated for mice and humans. Regional average structural covariance sex-bias and volumetric sex-bias were correlated to identify whether these features align in their direction of sex-bias.

**Results:**

We find that volumetric structural covariance is stronger in adult females than males for both wild-type mice and healthy human subjects: 98% of comparisons with statistically significant covariance sex differences in mice are female-biased, while 76% of such comparisons are female-biased in humans (q < 0.05). Regional covariance and volumetric sex-biases have weak inverse relationships to each other in both species: volumetrically male-biased regions contain more female-biased covariations, while volumetrically female-biased regions have more male-biased covariations (mice: *r* = -0.185, *p* = 0.002; humans: *r* = -0.189, *p* = 0.001).

**Conclusions:**

Our results identify a tendency for females to show stronger neuroanatomical covariance across species. These structural covariance sex differences are also partially related to regional sex differences in volume for both species, suggesting that stronger structural covariance in females could be an evolutionarily conserved feature - partially shaped by the same developmental influences that mediate volumetric sex-biases.

**Supplementary Information:**

The online version contains supplementary material available at 10.1186/s13293-025-00728-1.

## Abstract (plain language summary)

In any population, how two biological structures tend to vary together is called structural covariance, or anatomical correlations. For example, if individuals with a large structure A also show large structure B, then A and B are said to have structural covariance. In the brain, how closely two structures covary is hypothesized to reflect how much they have in common during development. Sex serves as a natural experiment for testing this hypothesis because parts of the brain that differ in size between males and females have undergone sex-biased developmental influences. Thus, if shared developmental influences shape structural covariance, then regions that show sex differences in their mean size should tend to show sex differences in their structural covariance. Using neuroimaging, we test this hypothesis in mice and humans by asking: are volume covariance sex differences present throughout the brain, where in the brain do they reside, and do these regions tend to show sex differences in mean volume? We show that structural covariance sex differences exist in both species, with females showing stronger structural covariance than males. Across mice and humans, regions with shared male influences tend to have stronger covariance in females and vice versa. Therefore,stronger volumetric structural covariance in females may be a trait conserved during evolution, and the factors that shape it may be shared with those that shape sex differences in regional anatomy.

**Supplementary Information:**

The online version contains supplementary material available at 10.1186/s13293-025-00728-1.

## Highlights

- Structural covariance is a potent readout of coordinated brain development, but hard to probe experimentally.
- We use sex differences as a naturally occurring test for developmental theories of structural covariance– adopting a cross-species approach for validation and translational benefit.
- Brain MRI reveals two conserved features of anatomical covariance across humans and mice: (i) tighter inter-regional coordination of brain development in females as evidenced by stronger volume covariance; (ii) a tendency for female-biased covariance to involve regions that are smaller in females– suggesting a previously unknown counterbalancing between these two distinct modes of sex-biased brain organization.
- These findings advance understanding of coordinated brain development and sex difference in a cross-species framework– facilitating future translational research on both topics.

**Supplementary Information:**

The online version contains supplementary material available at 10.1186/s13293-025-00728-1.

## Background

Structural covariance refers to the phenomenon in which variable biological structures in a population scale together across individuals. The degree of covariation between two structures is typically taken as evidence for how strongly they relate at some unmeasured levels of biology [1]. It is thought that observed anatomical covariances between regions in adulthood reflects their shared developmental influences in earlier life. This idea is based on published observations that higher inter-regional structural covariance tracks with stronger inter-regional coupling in connectivity [2, 3], gene co-expression [4, 5], and developmental tempos [6, 7]. A direct way to test this hypothesis would be to experimentally manipulate the developmental influences on multiple brain regions across multiple individuals and then estimate structural covariance across the group at a later time point. However, this experimental approach is technically challenging and could introduce unintended changes that confound interpretations of developmental effects on covariance formation.

Sex differences in brain organization serve as an alternative approach for testing the “shared influences” model of structural covariance. Experimental data have identified rodent brain regions with reproducible volumetric sex differences [8] through regional action of male-specific hormonal effects [9–15]. These volumetric sex differences include male-biased volume in the bed nucleus of the stria terminalis (BNST), olfactory bulb, medial amygdala, and female-biased volume in the anteroventral periventricular nucleus (AVPV) [9, 11–15]. Humans also show highly reproducible sex differences in regional brain anatomy [16, 17] that presumably reflect sex-biased regional brain development. Some of these biases share directionalities with mice, such as the male-biased BNST and medial amygdala [18]. If adult neuroanatomical covariances arise through an inter-regional sharing of developmental influences, then one would expect that volume covariance tends to be more sex-biased amongst brain regions that are sex-biased in mean volume than amongst those that are not. Thus, the study of sex-differences in neuroanatomical covariance not only sheds light on an understudied axis of sex-biased brain organization but also provides a naturally occurring probe for developmental models of covariance. To date, however, there is pronounced heterogeneity in results across those few studies that have tested for sex-biased structural covariance in humans [19–25] without comparisons to sex-differences in regional volume. Recent works in larger cohorts suggest cortical thickness covariance patterns are stronger in males while volume covariance patterns are stronger in females [26, 27]. To our knowledge, there are no published studies explicitly examining normative sex-biased neuroanatomical covariance in mice.

Here, we use cross-species structural magnetic resonance imaging (sMRI) to map sex-biased brain volume covariance and sex-biased volume. We test the hypothesis that these features are non-randomly correlated across the brain. Addressing this question requires computing sex differences in covariance between all pairs of regions within each species– which enables us to address several related questions including: (i) whether there is a tendency towards stronger volume covariance in one sex within each species, (ii) whether there is an inter-species differences in the strength of inter-regional neuroanatomical covariance, (iii) what specific brain regions and inter-regional pairs show sex-biased covariance in each species. Taken together, our work provides the first comparative analysis of sex-biased neuroanatomical covariance in humans and mice. Our results inform dominant developmental theories for the emergence of structural covariance and expand our comparative understanding of sex-biased mammalian brain organization.

## Methods

### Acquisition and processing of murine neuroimaging data

Our study includes structural MRI (sMRI) brain scans from 423 mice acquired at the Mouse Imaging Centre in Toronto. Scans were performed on the same 7T multichannel scanner with either an insert gradient (6 cm inner bore diameter magnet) or an outer gradient (30 cm diameter bore diameter magnet) (Agilent Inc., Palo Alto, CA). Mice were transcardially perfused using a standard protocol across all cohorts [28–30]. Brains were kept in the skull and fixed to avoid distortions during imaging. All animal procedures were approved by the ethics committees of their originating labs and the animal care committee at The Centre for Phenogenomics (AUP-0260 H) at the University of Toronto.

As the mouse data for this study was collected over 10 + years, the MRI pulse sequences were optimized over that period to increase the scanning throughput to enable 16 mice to be scanned in one session, and/or to improve resolution and increase gray/white matter contrast in each scan [29, 31]. The following three MRI sequences were used in overnight scans throughout the studies included here. (1) 3 brains scanned in parallel per session -- T2-weighted fast spin echo (FSE): TR = 325 ms, TE = 10 ms/echo for 6 echoes. The center of k-space is acquired on the 4th echo. Field-of-view (FOV) = 14 × 14 × 25 mm^3^. Matrix size = 432 × 432 × 780. Image resolution = 32 μm isotropic voxels. (2) 16 brains scanned in parallel per session (sequence 1***)*** -- T2-weighted 3D FSE: TR = 2000 ms, echo train length = 6, TE_eff_ = 42 ms. FOV = 25 × 28 × 14 mm^3^, matrix size = 450 × 504 × 250. Image resolution: 56 μm isotropic voxels. Oversampling in the phase encoding direction by a factor of 2 was applied to move ghosting artifacts from k-space discontinuity to FOV edges. FOV was cropped to 14 mm after image reconstruction. (3) 16 brains scanned in parallel per session (sequence 2) -- T2-weighted 3D FSE: TR = 350 ms, TE = 12 ms/echo for 6 echoes. Cylindrical 3D k-space acquisition. FOV = 20 × 20 × 25 mm^3^, matrix size = 504 × 504 × 630. Image resolution: 40 µmm isotropic voxels [32]. Of note, these sequences were evenly distributed between male and female mice.

Structural MRIs were registered and warped to an average study mouse template using deformation-based morphometry [33–37]. Log-transformed Jacobian determinants for each voxel were calculated and used to determine voxel volume differences between individual mouse brains with the averaged brain [38]. ROI volumes were calculated as the sum of volume differences for each voxel within the ROI. This process used the MAGeT algorithm [39, 40] and resulted in 336 unique brain regions from previously published atlases [41–45]. 255 of these regions were grey matter and included in the study.

The imaged mice consist of C57BL6J (*n* = 152) and C57BL6N (*n* = 271) wild-type controls from separate studies that each compared wild-type controls with mutations of a different autism-related risk gene [31]. For the purposes of this study, we only included wild-type cohorts which had at least 5 male and 5 female mice surviving a quality assessment procedure to flag and remove outliers. This procedure involved an initial visual quality control was performed to ensure accurate registration and segmentation, followed by an outlier detection process.

Regional volumetric measures for the full set of 423 sMRI scans was then subjected to batch control using ComBat (*sva* library in R) to correct for variability between strain and ASD gene cohort of origin [46–49]. Final animal characteristics are detailed in Table 1.

**Table 1:** Demographics for mouse sample

	Female	Male	Statistics
**Sample size**	211	212	
**Age (days)**			
Mean	62.0	62.6	F (1, 421) = 0.49, *p* = 0.44
SD	7.6	8.5	
Range	56–90	56–90	
**Background Strain**			
C57BL-6 J (12 cohorts)	132	139	X^2^ = 0.29, *p* = 0.59
C57BL-6 N (6 cohorts)	79	73	

### Acquisition and processing of human neuroimaging data

This study includes 436 human sMRI brain scans from the Human Connectome Project 1200 release. Scans were obtained using an MR750 3-T (General Electric) whole-body scanner (MP-RAGE-T1: TE 2.14 ms, TR 2400 ms, flip angle = 8°, FOV 224 × 224 mm2, scan time = 7:40 min, voxel size = 0.7 mm isotropic) with a 32-channel head coil (176 continuous sagittal slices with 256 × 256 in-plane matrix and 1 mm slice thickness). Additional recruitment procedures and acquisition parameters are detailed in the original publication [50, 51]. Information on how to obtain HCP data can be found here (https://www.humanconnectome.org/study/hcp-young-adult/document/wu-minn-hcp-consortium-restricted-data-use-terms). 1110 unique subject scans were visually inspected and removed if obvious registration and/or segmentation issues were detected. Euler numbers– indicators of brain topological reconstruction quality– were also measured for each scan using the image preprocessing steps described below [52]. Scans with FreeSurfer-estimated Euler numbers less than − 217 were excluded from further analyses [53]. From the remaining 1030 subject scans, we randomly selected one person per family, based on distinct mother ID and father ID, to yield 436 unique and unrelated subjects.

All remaining subjects underwent an outlier flagging process using maximal Cook’s distance. Briefly, pairwise linear models were created between all brain structures using mixed sex data. Subjects’ maximum Cook’s distances from all linear models are recorded. This served to identify whether a subject appeared to be unusually influential on the relationship between any pairwise correlations. A subject was deemed to have an unusually influential effect on pairwise relationships if its maximum Cook’s distance was greater than the standard cut off: 3 z-scores of the maximum Cook’s distance for all subjects. Such subject’s scan would be flagged for a secondary visual review. If a further review did not show abnormalities in the scan, it was kept in the data set. Final participant characteristics are detailed in Table 2.

The PreFreesurfer pipeline was used to preprocess T1-weighted structural MRI data (Glasser et al., 2013). Freesurfer 7.1.0’s [54] *recon-all* and *highres* commands were used to reconstruct and parcellate the cortex at the original data resolution [52, 55–68]. The pipeline can be downloaded here (http://surfer.nmr.mgh.harvard.edu/). Cortical volumes were extracted using the *mri_anatomical_stats* utility. 360 regions from the Glasser Human Connectome Project were generated using this procedure [69]. 359 regions from this atlas were used for subsequent analyses.

Subcortical and hippocampal segmentation was performed by first assigning one of 39 labels from the FreeSurfer ‘aseg’ feature to each voxel [61, 70]. 19 of these labels were gray matter structures and were included in subsequent analyses. Additional segmentations of sex-biased nuclei in the hippocampal subfield, amygdala sub-nuclei, and brainstem were made using FreeSurfer joint segmentation of these subfields [71–73]. Segmentations for classically sex-biased BNST and hypothalamic nuclei were made under a different atlas that is not available within FreeSurfer [74] (https://zenodo.org/record/3942115). Hypothalamic atlas labels were registered to the study’s average template, and deformation-based morphometry was applied to warp each subject’s image to the study’s template [75] (https://github.com/CoBrALab/optimized_antsMultivariateTemplateConstruction). The Jacobian determinant from this process was used to calculate the volume change from the template at each voxel in the region of interest (ROI). A summation of these changes within the ROI results in its volume measurement.

**Table 2:** Demographics for human sample

		Females	Males	Statistics
Sample size		238	198	F (1, 434) = 25.99, *p* = 5.143e-07
Age	*Mean*	29.4	27.6	
	*SD*	3.7	3.6	
	*Range*	22–36	22–35	
Education (in years) *	*Mean*	15.0	14.8	F (1,433) = 1.45, *p* = 0.23
	*SD*	1.8	1.7	
	*Range*	11–17	11–17	
Euler number	*Mean*	-53.7	-59.0	F (1,434) = 8.29 *p* = 0.004
	*SD*	18.4	20.6	
	*Range*	-126 to -16	-201 to -20	
Zygosity*	*Monozygotic*	72	37	X^2^ = 10.84, *p* = 0.004
	*Dizygotic*	66	49	
	*Not Twin*	99	111	

*1 subject did not have education information reported. 2 subjects did not have zygosity information reported. Statistics were only performed on subjects with available demographic data

ANOVA and chi-square tests of significant difference between groups (males vs. females). SD = standard deviation

### Comparing regional volume covariance between males and females in each species

To compare region of interest (ROI) covariance across sex in each species, we split the data for each species by sex and regressed age out of ROI volumes for both species using the following model:

$${\rm{ROI\_volume}}\; \sim \;\;{\rm{intercept }}\;{\rm{ + }}\;{\beta _{\rm{1}}}\;({\rm{ age }}){\rm{ + }}\;{\rm{\varepsilon }}$$

Residuals from this model were used to compute all pairwise inter-regional volume correlations within males and females. The means of these correlations across all pairwise relationships were directly compared between males and females using t-tests and reported 95% confidence intervals (CI). We then subtracted the male correlation matrix from the female to derive a measure of sex differences in correlation for all region pairs (with positive values indicating a larger correlation in males vs. females). The statistical significance of sex differences in correlation for each unique pair of regions was determined by repeating the above process 1000 times with sex being permuted across individuals for each iteration. This procedure yielded a vector of 1000 null values for each pairwise correlation sex differences and we derived empirical p-values for these observed sex differences against these nulls. Empirical p-values were corrected for multiple comparisons across edges using the False Discovery Rate (FDR) [76, 77] correction with q (the expected proportion of falsely rejected nulls) being set at 0.05.

### Examining the relationship between sex differences in volume covariance and sex differences in regional volume

Sex differences in regional volume were estimated as follows: ROI and total grey matter tissue volumes (TTV) were z-scored across individuals, then input into the following models to estimate the effect of sex on the mean volume of each brain region (given by the β_1_ coefficient in the model below):

$$\eqalign{& {\rm{ Mice: ROI\_volume}} \sim {\rm{ intercept + }}\; \cr & {{\rm{\beta }}_{\rm{1}}}({\rm{ Sex: male }}\;{\rm{ vs }}\;{\rm{ female }}){\rm{ + }}\; \cr & {{\rm{\beta }}_{\rm{2}}}({\rm{ age }}){\rm{ + }}\;{{\rm{\beta }}_{\rm{3}}}({\rm{ TTV }}){\rm{ + }}\;{{\rm{\beta }}_{\rm{4}}}({\rm{ Background\ Strain}})\; + \;{\rm{\varepsilon }} \cr} $$

$$\eqalign{& {\rm{ Human: }} \cdot {\rm{ROI\_volume}}\; \sim \;{\rm{intercept }}\; \cr & {\rm{ + }}\;{{\rm{\beta }}_{\rm{1}}}({\rm{ Sex: }}\;{\rm{ male }}\;{\rm{ vs }}\;{\rm{ female }})\;{\rm{ + }} \cr & \;{{\rm{\beta }}_{\rm{2}}}({\rm{ age }})\;{\rm{ + }}\;{{\rm{\beta }}_{\rm{3}}}({\rm{ TTV }})\;{\rm{ + }}\;{{\rm{\beta }}_{\rm{4}}}({\rm{Euler \ Number) }}\;{\rm{ + }}\;{\rm{\varepsilon }} \cr} $$

Positive beta coefficients in the model indicate male-biased regional volumes. p-values associated with the β_1_ coefficients from each of these models were corrected for multiple comparisons across the number of brain regions in each species using FDR with q < 0.05.

The relationships between covariance and volumetric sex differences were assessed using several complimentary approaches. First, we selected three classically sex-biased regions in mice– the BNST, medial amygdala, and olfactory bulb– and asked if there are any covariance sex differences among these pairings. Statistical significance was calculated using the previously described permutation pipeline and Bonferroni corrected for the total number of comparisons made within this analysis (*p* = 0.05). Second, we used the full correlation sex differences matrix in each species to estimate the mean sex differences in volume correlation per brain region (averaging the sex differences in its correlation with all other regions) - once using all pairwise correlation sex differences, and again using just those pairs deemed statistically significant in covariance sex differences through permutation testing. We examined the distribution of these properties across the brain of each species and noted those brain regions showing both sex differences in mean volume and sex differences in volume covariance. Third, we used network visualization to specify sets of brain regions showing prominent sex differences in anatomical covariance. Specifically, we identified all region pairs with statistically significant covariance sex differences, converted these pairings into a graph with nodes (regions) and edges (covariance sex differences), and visualized the largest connected components of these graphs in each species to determine their contents and any included nodes that also show sex-biased volume. To capture a similar number of nodes across species for these graphical representations we examined the two largest connected components in mice and the single most connected component in humans. Fourth, we tested if inter-regional variation in the mean sex difference in volume covariance per brain region was correlated with inter-regional variation in the effect size of volumetric sex differences (β_1_ coefficients). These correlations were run twice in each species– once using the regional means for the absolute sex differences in correlations and once using regional means for signed sex differences in correlations. The absolute value test asks if regions with a larger volumetric sex bias tend to show larger sex-differences in their inter-correlations - irrespective of the “direction” (i.e. male-biased vs., female-biased) of these differences. In contrast, the signed test asks if regions with a more strongly biased volume in one sex versus the other tend to show similarly signed sex differences in their covariance (i.e. regions with male-biased in volume tend to show male-biased in their covariance). The statistical significance of these correlations between regions’ sex differences in covariance and regional sex differences in volume was assessed by comparing observed correlations with a distribution of 1000 null correlations from permutations of sex within species (performed on the NIH HPC Biowulf cluster -- http://hpc.nih.gov). These global correlations between sex-biased regional volume covariance and sex-biased regional volume represent the core brain-wide test of our primary motivating hypotheses based on developmental theories for structural covariance: that brain regions exposed to sex biased influences on their volume in development (manifest as sex-biased mean volume in adulthood) would therefore be expected to show more prominent sex-biased covariance than brain regions lacking a sex difference in volume.

### R versions and packages

All analyses presented in this paper were performed using R version 4.2.3 unless Biowulf handled the computation, in which case R version 4.2.1 was used (R Core Team, 2023). Packages used for all analyses can be found in the references section. [78–91]. Data cleaning and analyses codes can be found at github.com/phamlk/cross-species-covariance-sex-differences.

## Results

### Mean structural covariance is slightly stronger in females than males for both mice and humans

**Fig. 1:**
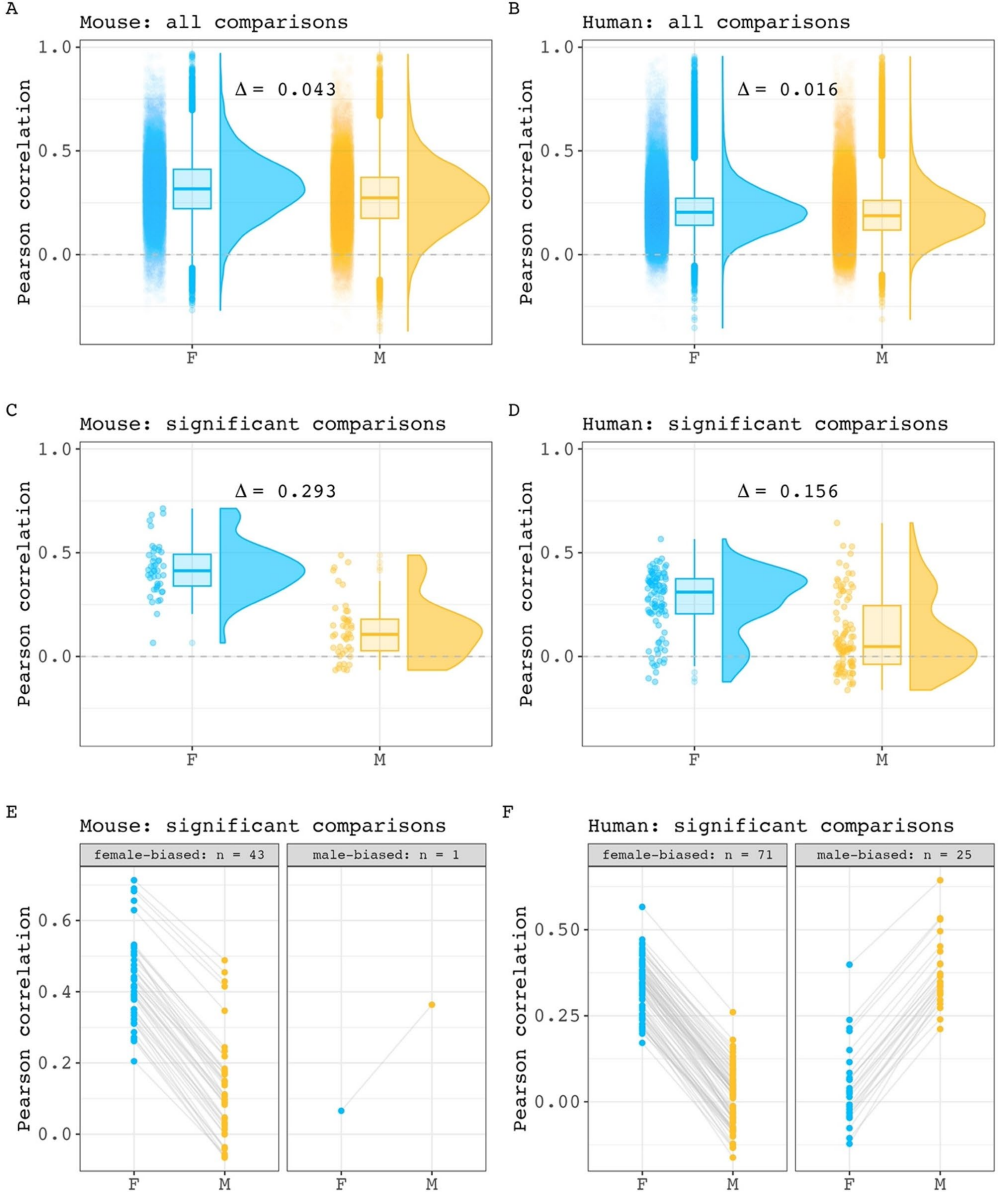
Inter-regional volume correlation distributions across sex in mice and humans. **A**, **B**) Comparison of all within sex correlation values for mouse (**A**) and human (**B**) (Δ mouse = 0.043, 95% CI: 0.041–0.045. Δ human = 0.016, 95% CI: 0.015–0.017). Each point represents a pairwise correlation value. Box plots and density plots are shown for distribution visualizations. **C**,** D)** Comparison of within-sex correlation values for region pairings with statistically significant covariance sex differences in mouse (**C**) and human (**D**) (Δ mouse = 0.293, 95% CI: 0.235–0.353. Δ human = 0.156, 95% CI: 0.108–0.204). **E**,** F)** Pairwise visualization of comparisons with significant sex differences in mouse (**E**) and human (**F**). Each point represents a pairwise correlation in either male or female. A connecting line between two points are shown to connect a pairwise correlation value in one sex and with its equivalent pairing in the other sex

For both species, we first compared the means of all correlations in each sex prior to identifying specific interregional pairs with large covariance sex differences. Our comparison showed that mean interregional covariance is stronger in females than males, with a small magnitude for the mean between sex difference (Δ) in covariance (Fig. 1 A - B, Δ mice: 0.043, 95% CI: 0.041–0.045; Δ humans: 0.016, 95% CI: 0.015–0.017). After correction for multiple comparisons across region pairs, we identified 44 pairs of regions with statistically significant sex-biased covariance in mice (0.14% of all pairwise relationships in the mouse brain; 43 stronger covariance in females) and 100 such pairs in humans (0.10% of all pairwise relationships in the human brain: 71 stronger covariance in females). As expected, the mean of covariance strengths for these pairs differed between the sexes for both species - with a larger magnitude than was seen when considering all pairs (Fig. 1C - F, Δ mice: 0.293, 95% CI: 0.235–0.353; Δ humans: 0.156, 95% CI: 0.108–0.204). Of note, the average within-sex correlation was consistently higher in mice than in humans (all comparisons: 0.275 in male mice, 0.196 in male humans. 0.318 in female mice, 0.212 in female humans; significant comparisons: 0.129 in male mice, 0.106 in male humans. 0.422 in female mice, 0.261 in female humans). The full within-sex structural covariance matrices and lists of pairwise covariance sex differences can be found for each species in Supplemental Figs. 1–1, 1–2 and Supplemental Tables 1–1, 1–2. As part of these analyses, we also tested the expectation that sex differences in volume correlation are more pronounced in region pairs showing weaker within sex volume correlation (as simulated in Supplemental Fig. 1–3). We confirmed the presence of an inverse relationship between covariance strength and covariance sex differences when considering pairings at various correlation sex differences significance thresholds. The results of these analyses are provided in Supplemental Fig. 1–4. Taken together these results indicate that regional volume covariance is stronger in mice than humans for both sexes, and that within each species, females tend to show stronger structural covariance than males. For both species, these sex-differences in structural covariance are statistically significant for a small subset (< 0.15%) of all possible inter-regional pairings in each species, with the largest sex differences occurring between those inter-regional pairings that show weaker structural covariance in each sex.

### Cross-brain analysis reveals a weak association between interregional sex differences in volume covariance and sex differences in regional volume

**Fig. 2:**
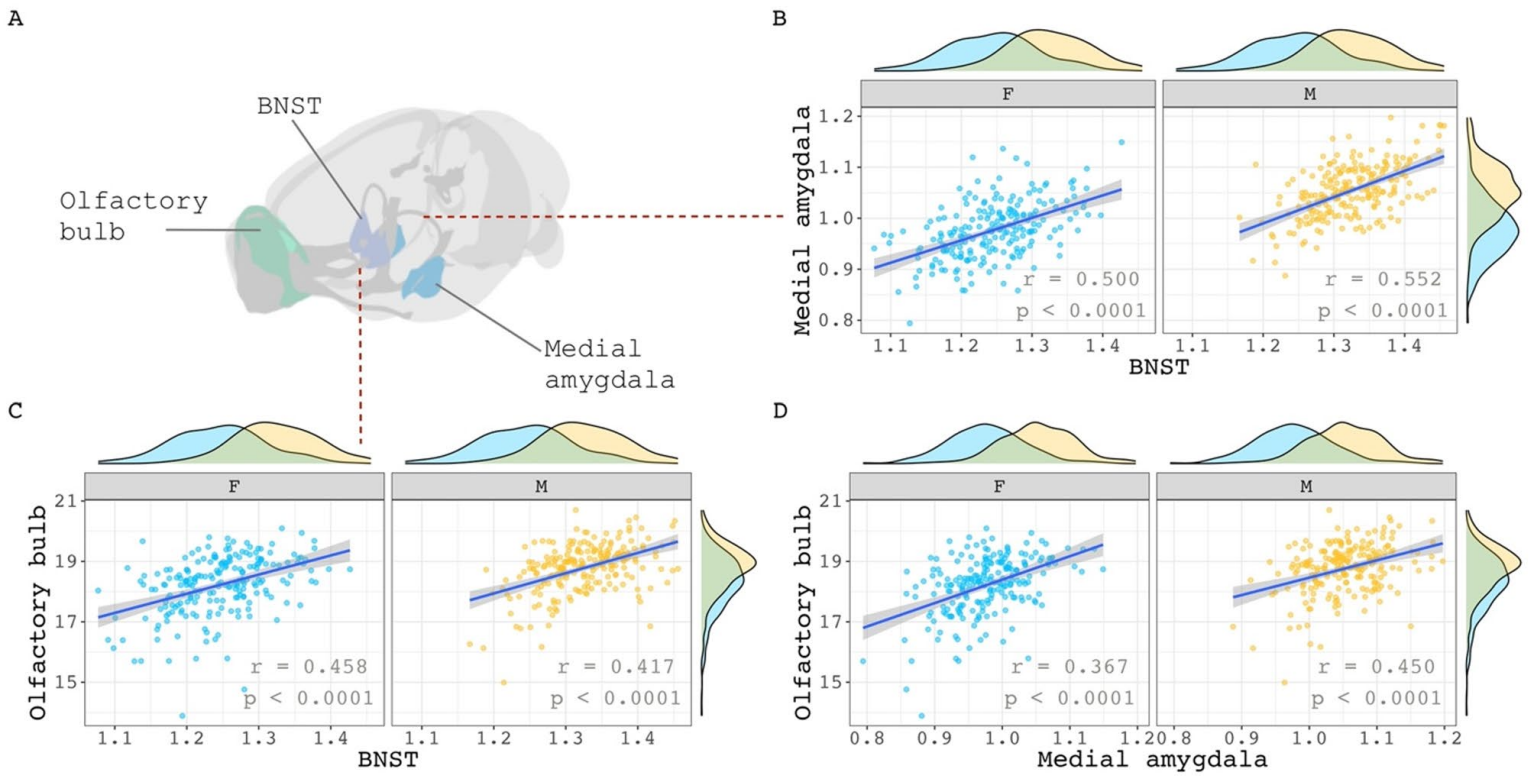
Within sex correlations between classic sex-biased regions in the mouse. **(A)** Visualization of three volumetrically sex-biased regions: olfactory bulb, BNST, and medial amygdala. **B**,** C**,**D)** Sex-specific correlations of the medial amygdala with BNST (**B**), olfactory bulb with BNST (**C**), and olfactory bulb with medial amygdala (**D**). Sex-specific means of each region were added to their residuals before correlation calculations began. This was done to maintain volumetric sex differences in volume distribution for visualization purposes. Correlation sex differences for all three pairs were not statistically significant (*p* = 1.0)

We took several complementary approaches to examining the relationship between regional sex differences in brain volume covariance and regional sex differences in brain volume. First, we selected 3 classical regions with the largest and best-replicated sex differences in volume in mice– BNST, olfactory bulb, medial amygdala– and examined sex differences in covariance between these structures. None of the region pairs showed statistically significant sex differences in covariance (Fig. 2B-D: medial amygdala– BNST: Δ = 0.053, *p* = 1.0; olfactory bulb– BNST: Δ = 0.041, *p* = 1.0; medial amygdala– olfactory bulb: Δ = 0.083, *p* = 1.0).

**Fig. 3:**
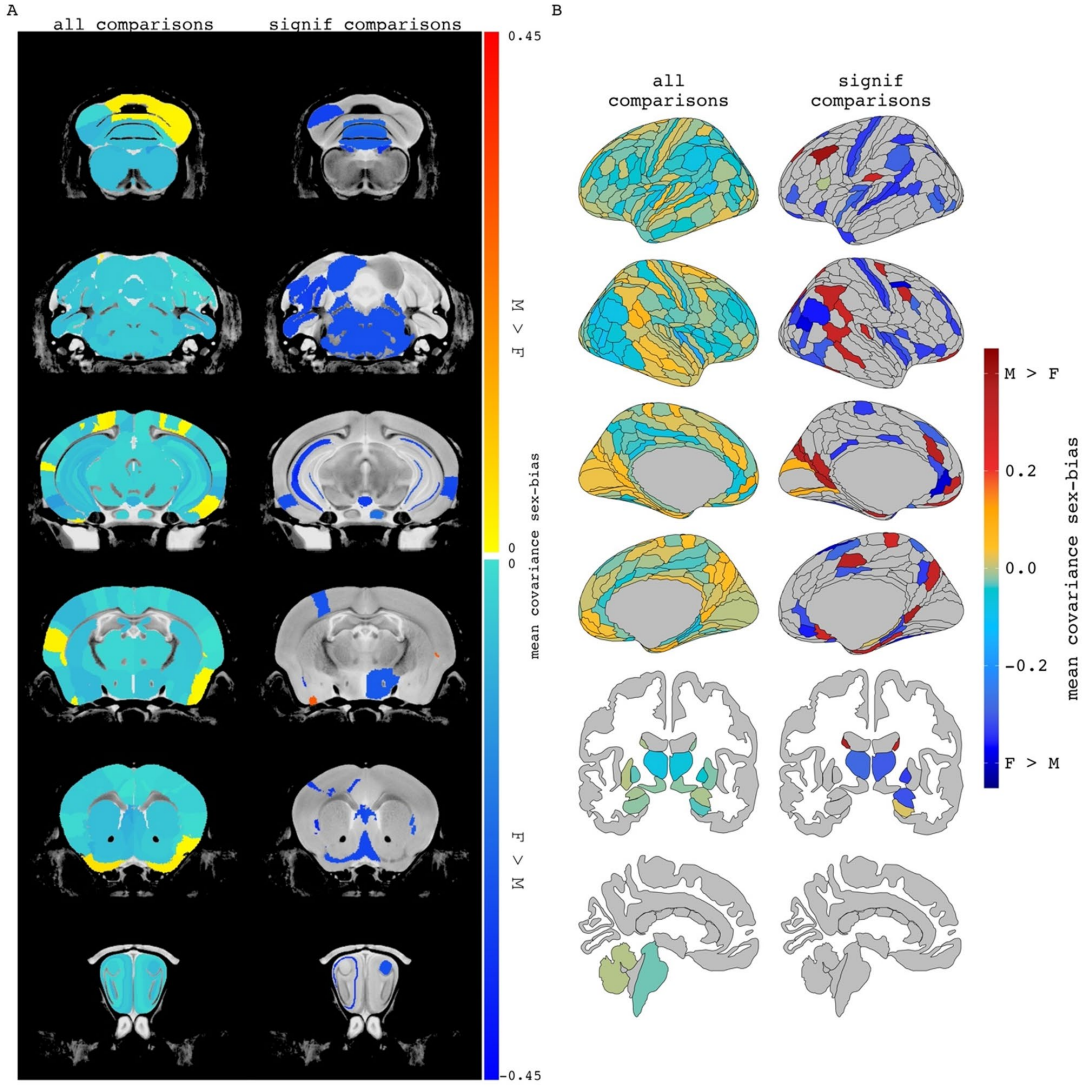
Regional mean covariance sex differences in mice and humans. Mean covariance sex differences in mouse (**A**) and human (**B**) when averaging a region’s covariance sex difference across all pairwise comparisons (all comparisons) or only pairwise comparisons with statistically significant covariance sex differences (significant comparisons). When including all pairwise comparisons, 89% of mouse regions had mean covariance sex differences which were stronger in females. For humans, averaging all pairwise covariance sex differences per brain region resulted in 68% of human regions with female-biased mean covariance. When including only comparisons significant for covariance sex differences, 96% of the included mouse regions and 70% of the human regions were female biased. The magnitude and direction of sex-bias of left and right regions generally correlate when considering all sex differences results (mice: *r* = 0.57; humans: *r* = 0.48)

Second, we expanded our analyses to characterize the relationship between sex-biased volume and sex-biased volume covariance throughout the brain of each species more broadly. To contextualize these analyses, we projected all observed sex-differences in volume covariance into anatomical space by computing the mean signed sex-difference in volume covariance for each region and visualizing the distribution of this regional value across the brain of each species (Fig. 3A, B– left columns). Re-computing these maps using information for just those region pairs with statistically significant sex differences in covariance (see Methods) highlighted several brain regions in each species with significant cumulative sex-differences in volume covariance with the rest of the brain (Fig. 3A, B -- right columns). In mice, these regions had almost exclusively stronger covariance in females and included the infralimbic area, medial parietal association cortex, and the pons. Regional covariance sex differences were also more often female-biased in humans and included regions such as the auditory complex 4, the primary sensory cortex, and the primary motor cortex. However, in humans, we also observed regions with significantly male-biased sex-differences in regional structural covariance, including the area prostriata and the premotor eye field. Qualitatively some of the regions highlighted by these analyses also showed sex-differences in mean volume [e.g. mice: mamillary body, mouth primary somatosensory area, claustrum (female-biased mean volume); BNST, olfactory bulb, CA3 pyramidal region (male-biased volume) / humans: primary sensory cortex, cingulate regions (supplementary and cingulate eye field, ventral area 24d) (female-biased volume); hypothalamus, amygdala, posterior insular region 1 (male-biased volume). The full lists of mean regional covariance sex differences in each species can be found in Supplemental Tables 3 − 1, 3 − 2.

**Fig. 4:**
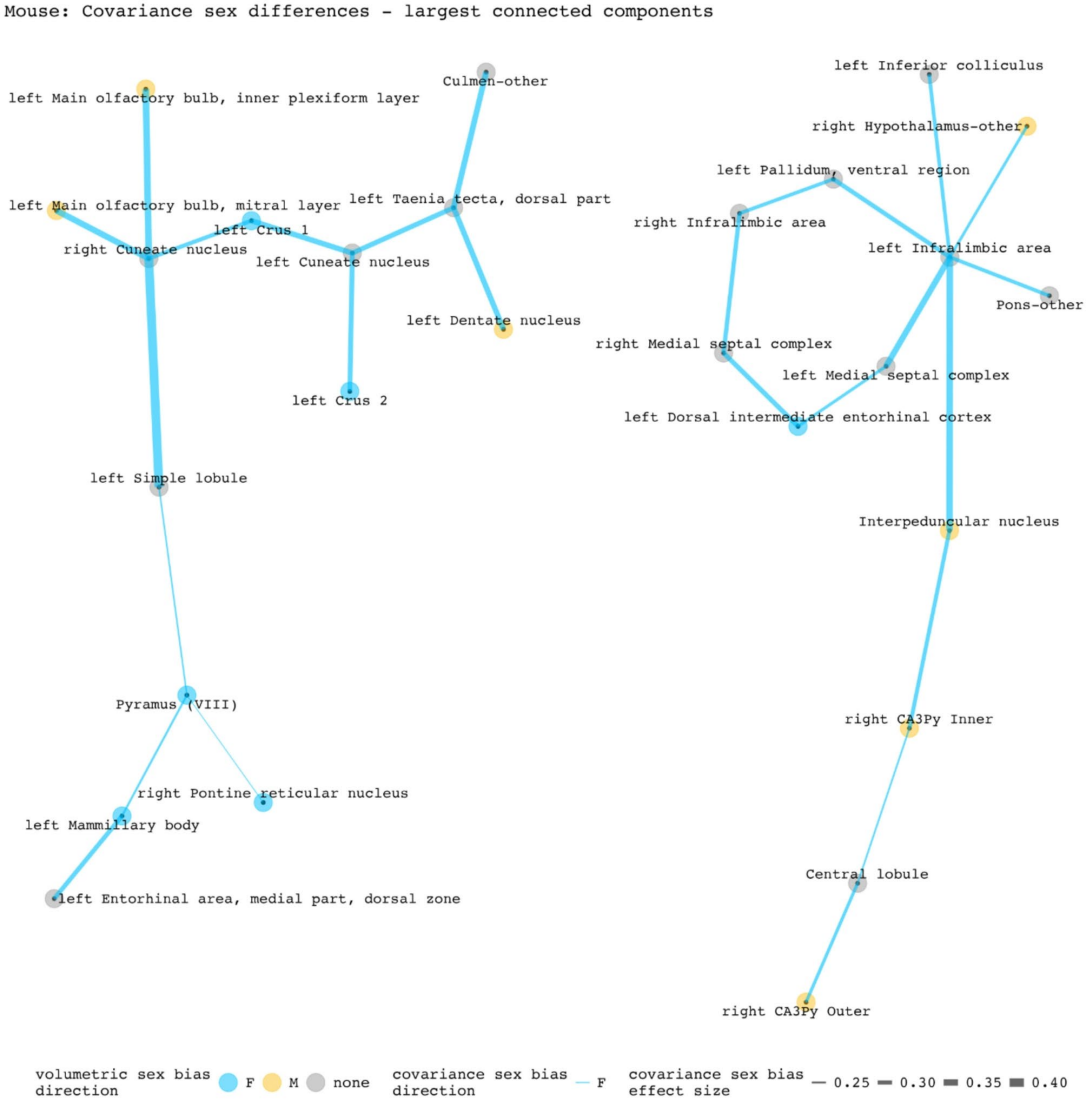
Statistically significant covariance sex differences in mice (nodes and edges of top 2 connected components). The first component (left side) is centered by the right cuneate nucleus and involves 14 regions/13 covariance pairs. The second component (right side) is centered on the left infralimbic area and involves 13 regions/13 covariance pairs. Both components contain only female-biased edges. Of the 16 edges involving volumetrically sex-biased regions in these components, 13 are between a volumetrically sex-biased region and a region without any volumetric sex bias; 3 are between two volumetrically sex-biased regions

**Fig. 5:**
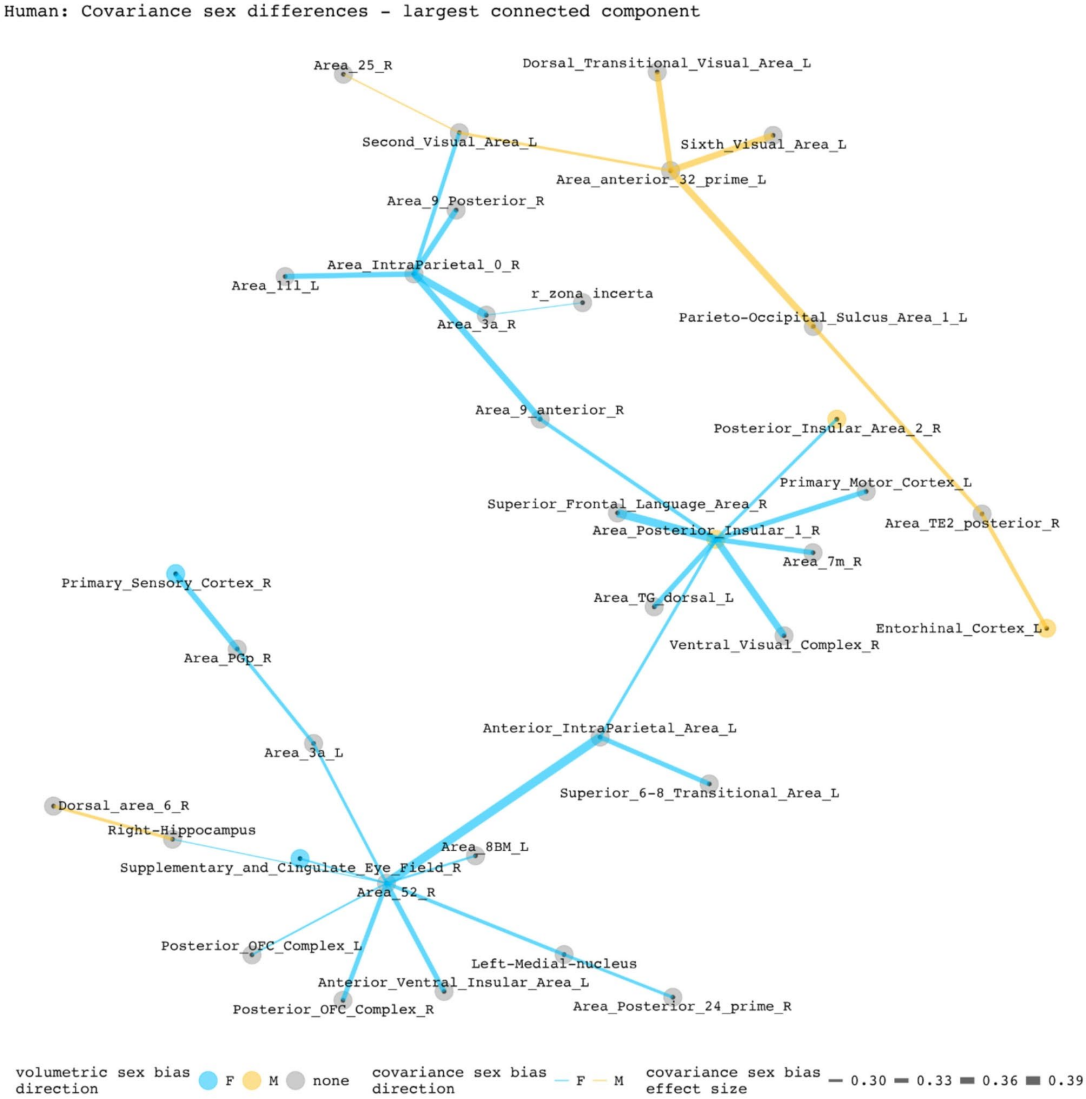
Statistically significant covariance sex differences in humans (nodes and edges of the most connected component). This component is centered by two regions: right parainsular area 52 with 7 connecting nodes and right posterior insular area 1 with 8 connecting nodes. Of the 33 edges included in this component, 8 are male-biased. Of the 35 regions included in this component, 3 have volumetric sex differences (right posterior insular area 1, right posterior insular area 2, right supplementary and cingulate eye field). Except for the female-biased covariance relationship between the right posterior insular area 1 and the posterior insular area 2, all sex-biased edges involving a volumetrically sex-biased region (9 in total) are paired with a volumetrically non-sex-biased region

To disentangle individual inter-regional pairs from these regional summaries of Fig. 3, and to further assess the involvement of volumetrically sex-biased regions in sex-biased covariance patterns, we generated graphs containing all inter-regional pairs with statistically significant sex-biased volume covariance in each species (regions as nodes and sex-biased covariance relationships as edges). Figures 4 and 5 represent the largest connected components of these graphs for mice and humans. In mice, the largest components are centered around the right cuneate nucleus and the left infralimbic area. All connecting covariance sex-biased edges are female-biased. Of the 27 regions involved in these components, 7 volumetrically sex-biased regions are distantly associated with the central nodes (nodes with 3 or more associated edges through intermediary connections). These regions include the male-biased hypothalamus and CA3 pyramidal regions, and the female-biased mamillary body and the cerebellar crus regions. The largest connected component in humans contains 35 regions and is mainly centered around the right posterior insular area 1, a volumetrically male-biased region, and the right parainsular region area 52. Approximately 80% of this component’s edges are female-biased. Only 2 other volumetrically sex-biased regions are represented in this component (supplementary and cingulate eye field; primary sensory cortex– both are female-biased).

**Fig. 6:**
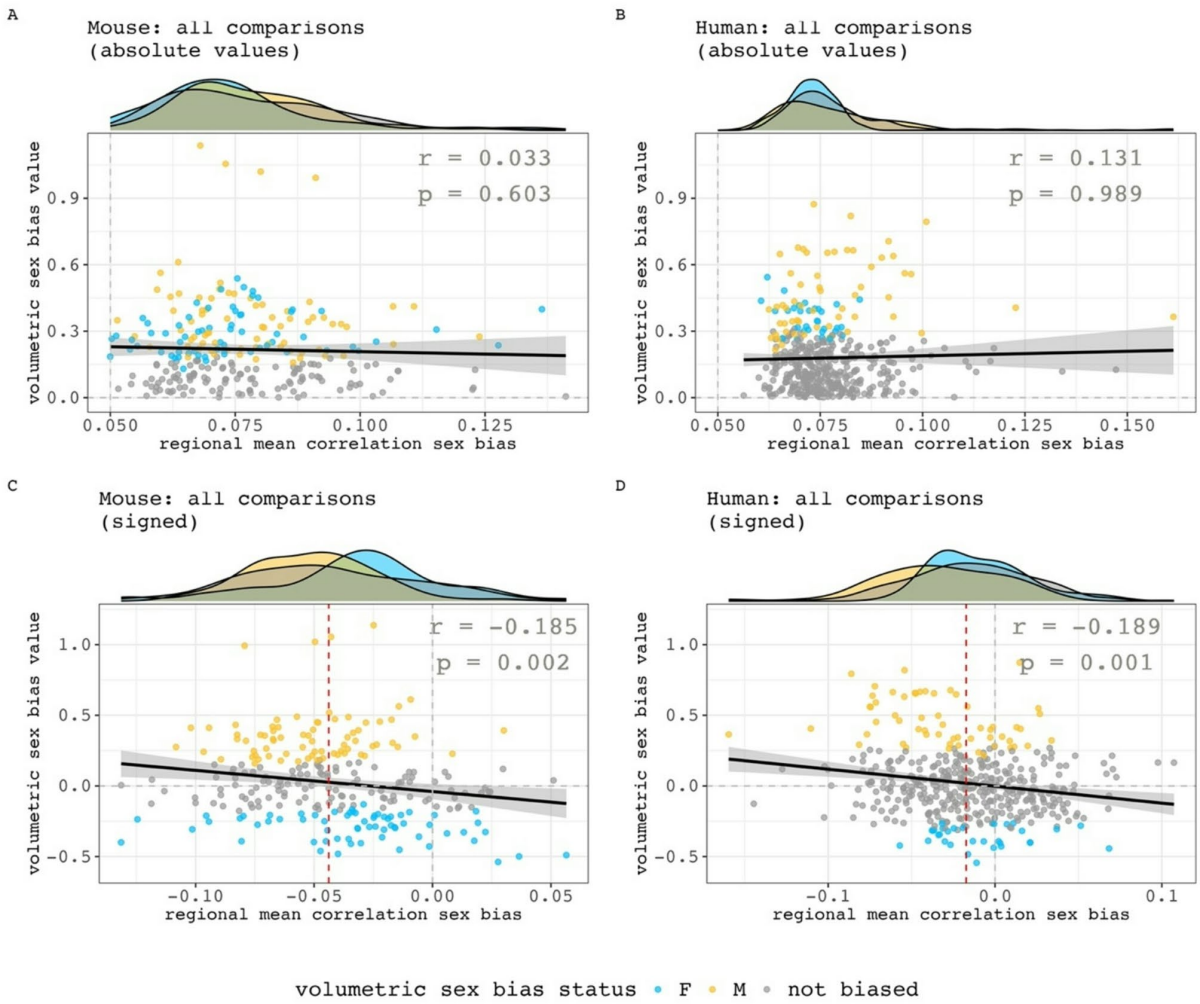
Relationships between the sex-biased in regional volumetric and regional volume covariance. **A**,** B)** Relationships between absolute values in mean regional correlation sex bias and volumetric sex biases in mouse (**A**) and human (**B**). **C**,** D**) Relationships between signed values in mean regional correlation sex bias and volumetric sex biases in mouse (**C**) and human (**D**). Negative values are female biased in both axes. Marginal density plots represent the regional mean correlation sex-bias distributions of different volumetric sex-bias categories (female-biased, male-biased and not significantly sex-biased). For the signed analyses in both species, there is a statistically significant, but weak negative correlation between sex-differences in volume and sex-differences in volume covariance (inset statistics). Thus - in both species - regions of significantly male-biased volume more often show female-biased volume covariance and vice versa for regions of significantly female-biased volume. However, note that regions with the largest volumetric sex-bias values do not have the largest mean correlation sex-bias– their values are concentrated closer to the median regional mean correlation sex bias (red dashed line).

Finally, we sought to quantitatively test– within both species - if regional variation in the magnitude of sex-biased volume covariance was related to regional variation in the magnitude of sex-biased mean volume. These analyses were repeated twice within each species - once using absolute values and once using signed values for mean regional sex differences in volume covariance (absolute inter-regional sex differences in covariance for computing regional means and absolute sex differences in volume) for all available regions. The absolute values analysis shows no evidence of an association between regional volumetric and mean covariance sex-bias effect size in both humans and mice (Fig. 6A, B, mice: *r* = 0.03, *p* = 0.68; humans: *r* = 0.13, *p* = 0.98). However, the signed analyses revealed that both species show a weak yet statistically significant inverse relationship between regional covariance and volumetric sex-bias directions (Fig. 6C, D, mice: *r* = -0.19, *p* = 0.002; humans: *r* = -0.19; *p* = 0.001). Specifically, in both species, regions of significantly male-biased volume more often show female-biased volume covariance and vice versa for regions of significantly female-biased volume.

Taken together, these analyses help to localize sex-differences in volume covariance within the brains of humans and mice and further specify the spatial relationship between this phenomenon and accompanying sex-differences in regional volume. We find several regions of prominently sex-biased volume covariance in each species– highlighting the infralimbic area, medial parietal cortex, and pons in mice and auditory complex 4, the primary sensory cortex, and primary motor cortex in humans. Some of these regions overlap with regional sex-differences in volume– highlighting brain areas that show two forms of sex-biased organization (e.g. mamillary body, mouth primary somatosensory area, claustrum in mouse; hypothalamus, amygdala, and posterior insular region 1 in humans). Although significant sex differences in anatomical covariance are not seen amongst major classical foci of sex-biased volume in the mouse brain, there is a subtle yet significant inverse association between sex differences in volume and volume covariance across both the murine and human brain.

## Discussion

In this study, we utilize sex as a natural experiment to ask whether developmental programming affects structural covariance formation within humans and mice. Our results provide a systematic survey of sex-biased neuroanatomical covariance and their conservation across species. We consider each of our main findings below.

First, en route to estimating sex-differences in structural covariance across species, we observe that mice show stronger neuroanatomical covariance than humans. This observation might reflect the combined action of several species’ differences. Greater genetic and environmental variability across humans compared to inbred laboratory mice likely translates into weaker coordination of anatomical variation within human brain. Greater neuroanatomical covariation in mice may also track with the gross brain size differences between species– larger vertebrates tend to show greater phenotypic variability [92] and inter-regional covariance strength likely drops off with the greater inter-regional distances within the human compared to the murine brain [4, 93]. Species differences in overall neuroanatomical covariance strength could coincide with substantially longer lifespan in humans than mice given evidence of age-related decreases in neuroanatomical covariance within humans [94].

Second, we replicate prior reports of female-biased volume covariance in the human brain [23, 26] and reveal that this is also evident in the mouse brain. This species convergence suggests that female-biased neuroanatomical covariance may be an evolutionarily conserved feature, though studies in other species are required to verify this. Many previously proposed candidate mechanisms for sex-biased anatomical covariance cannot parsimoniously account for the female-biased covariance observed in both species. Although sex-differences in overall brain-size would predict stronger anatomical covariation in female humans (given larger brain size in males)– this explanation could not account for sex-biased anatomical covariation in mice as they do not have overall brain size sex differences [18]. Similarly, although greater anatomical variability in human males could explain stronger anatomical covariation in human females (if male-biased anatomical variance in humans reflects greater developmental noise that is uncorrelated between regions), this explanation would not apply in mice– which do not show the prominent sex-bias in neuroanatomical variability evident in humans [18, 25, 26]. A larger human study also suggests that biological variation is a poor predictor of covariation strength: although subcortical volumes and cortical thickness are more variable in males, only cortical thickness correlations were higher in males [26]. The most parsimonious hypothesis for our findings would be that the tendency towards female-biased neuroanatomical covariance in humans and mice reflects a genetic and hormonal aspects of sex shared between species and capable of shaping inter-regional anatomical covariance. For example, females are biallelic for X-linked gametologs and males are hemizygous for X- and Y-member of each gametolog pair in both species. Given this, regionally specific functional divergences of X- vs. Y-gametologs [95] could represent a male-specific source of inter-regional divergence in neurobiological organization operative in both species, leading to stronger inter-regional covariance in females.

Third, we profile the regional distribution of sex-biased volume covariance in each species and probe how it relates to sex differences in mean regional volume. As an initial targeted test for the idea of coordinated sex-differences in brain volume and covariance– we focused on 3 canonically sex-biased regions in the mouse brain (BNST, medial amygdala, olfactory bulb) and did not find sex-biased volumetric covariance amongst these regions. Thus, male-specific processes driving highly reproducible male-bias in these regions’ mean volumes [9, 12, 15] do not appear to open male-specific sources of covariation between regions. However, we cannot rule out that these regions have sex-specific volume covariation sources that counterbalance each other or are hidden by dominantly shared sources of inter-regional volume covariation between the sexes.

Fourth, while no covariance sex differences were identified among classical regions of volumetric sex bias, broadening the search for sex-biased volume covariance identified multiple brain regions showing differential brain wide volume coupling between sexes. Echoing the female-bias volume covariance found in our global analyses, regional sex-biased covariance hotspots were almost exclusively female-biased in mice and mostly female-biased in humans. Regions with significant sex-bias in covariance in mice were the infralimbic area, medial parietal association cortex, and the pons (stronger in females); in humans, these regions include the auditory complex 4, the primary sensory cortex, the primary motor cortex (stronger in females), the area prostriata, and the premotor eye field (stronger in males). Some of the brain regions showing prominent sex-biased covariance are also volumetrically sex-biased in our study, such as the mamillary body and claustrum in mice and the posterior insular region 1 and hypothalamus in humans. These regions offer high-priority targets for follow-up mechanistic analyses and for probing how sex-biased anatomical organization of the brain might relate to brain function.

Fifth, to identify specific covariance sex differences that underpin these regional patterns, we visualized networks of significant covariance sex differences in mice and humans, flagging large sets of brain regions interlinked through sex-biased volume covariance. Some of the pairings in these networks have been shown through tract tracing and diffusion tensor MRI studies to be physically connected [96–99]. These findings help motivate challenging experimental studies that will now be needed to determine the drivers for sex-differences in neuroanatomical covariance, such as sex differences in structural connectivity.

Finally, while we define some instances of overlap between sex-biased volume covariance and sex-biased volume, we show that these features are not strongly related across the brain. This dissociation implies that processes regulating sex-biased volume and sex-biased covariance development are largely dissociable. However, we do observe a weak negative correlation between signed sex differences in brain volume and brain volume covariance– mostly driven by a tendency for volumetrically male-biased regions to show female-biased volume covariance. These results suggest that the influence of sex on a brain region partially decouples its growth trajectory from other brain structures. Growth decoupling of several brain regions via sex could effectively lower their overall morphometric integration and result in a more modular mode of brain organization [100]. Thus, in mice and humans, weaker structural covariance may reflect more modular brain organization in males compared to females. We hypothesize that higher modularity may be the result of a sexual selection process that simultaneously produces volumetric sex bias and increases diversity in brain integration patterns in males [16, 101]. Increased integration diversity due to sex could increase the likelihood for pathologic integration patterns to appear in males and account for the earlier emergence of neurodevelopmental conditions in males relative to females [102]. If this is true, an individual’s deviation from a sex-specific structural covariance norm would be predicted to correlate with other neurodevelopmental outcomes [103].

Our findings should be considered with several limitations. First– we focused on brain volume as a phenotype that can be compared across species by sMRI. However, sMRI can only resolve sex differences in volume and covariance at or above the lower limit of its voxel dimensions (here, ~ 40 µm^3^ in mice and ~ 1 mm^3^ in humans). Sex differences in structures indiscernible by contrast in T2-weighted MRI are also not detectable. Second, there are numerous other brain properties beyond volume that could be measured by sMRI in humans and mice (e.g. regional myelin content and cortical thickness). We therefore cannot assume our volumetric findings will generalize to other properties. Third, we estimate covariance cross-sectionally in adulthood datasets in each species. As such, we cannot speak to age-related variations of sex-biased anatomical covariance. Fourth, we note that there is not straightforward convergence between brain regions that show prominent sex-differences in volume covariance in humans versus mice (Figs. 4 and 5). However, theoretical and methodological approaches for inferring homologies between human and mouse brains are rapidly evolving [18, 104] and could be applied in the future to formally assess regional species convergence. Finally, our study design is purely observational, and we cannot dissect potential mechanistic or functional bases for observed sex differences in covariance. This is especially true given the maximum magnitude in correlation differences is small and there are few covariance sex differences overall. In mice, these differences could ultimately be related to sex-biased chromosomal and gonadal influences on brain development. Such influences are likely operative in humans but will certainly be interwoven with co-occurring gendered influences stemming from the external environment. Transgenic models like the Four Core Genotypes could explain how covariance sex differences are formed [8, 105]. These experiments would expand our understanding of how sex affects brain organization and how developmental programming can drive covariance formation.

Notwithstanding the limitations above, our study design was able to use sex differences as an informative probe for theories regarding the developmental bases of neuroanatomical covariance, and– en route to doing so - provide a thorough parallel mapping of global and inter-regional sex differences in brain volume covariance for the human and murine brain. We validate existing human results that show females have a tendency towards stronger anatomical covariance than males, and show the same phenomenon exists in mice. We provide a fine-grained delineation of brain systems that show sex-biased covariance in each species. Our finding that structural covariance sex differences minimally involve volumetrically sex-biased structures supports the viewpoint that the sex influences likely have largely uncoordinated effects across brain regions and highlights structural covariance as a novel axis of sex-biased brain organization warranting further study. Additionally, the cross-species approach used here allows us to articulate the potential impacts of stochasticity and variation on structural covariance, emphasizing the importance of synchronizing methodologies across species for gaining new insights into brain biology and increasing the translatability of animal findings [106].

## Conclusions

In this study, we use sex as a natural experiment to probe whether structural covariance formation depends on shared developmental influences in mice and humans. Because males and females have different dominating developmental influences, the presence of structural covariance sex differences in our study suggests that shared developmental factors within each sex contribute to sex-specific structural covariance patterns. Similarities in structural covariance sex differences trends across species suggest that female-biased structural covariance may be an evolutionarily conserved feature. Validating this statement will require studying the same phenotype in closer animal relatives of humans. Studies of structural covariance using animal models that can differentiate sex hormones and chromosomes effects will also be needed to identify the specific aspects of sex that influence structural covariance formation.

## Electronic supplementary material

Below is the link to the electronic supplementary material.

Supplementary Material 1: Supplemental Table 1-1. All pairwise covariance sex difference results in mice. Differences significance status is defined in the “signif” and “signif_adj” columns, corresponding to whether the difference is significant after permutation testing or after permutation testing and multiple comparisons corrections.

Supplementary Material 2: Supplemental Table 1-2. All pairwise covariance sex difference results in humans. Differences significance status is defined in the “signif" and ”signif_adj" columns, corresponding to whether the difference is significant after permutation testing or after permutation testing and multiple comparisons corrections.

Supplementary Material 3: Supplemental Table 3-1. All regional mean covariance sex differences in mice. Signed values are indicated as “meanCov_signed”. Absolute values are indicated as “meanCov_absolute”.

Supplementary Material 4: Supplemental Table 3-2. All regional mean covariance sex differences in humans. Signed values are indicated as “meanCov_signed”. Absolute values are indicated as “meanCov_absolute”.

Supplementary Material 5: Supplemental Figures 1-1, 1-2, 1-3, 1-4.

## Data Availability

The datasets generated and/or analysed during the current study are available in the following Github (github.com/phamlk/cross-species-covariance-sex-differences).

## References

[1] Galton F. Co-relations and Their Measurement. Proceedings of the Royal Society of London. 1888:135– 45; https://galton.org/essays/1880-1889/galton-1888-co-relations-royal-soc/galton_corr.html

[2] Andrews TJ, Halpern SD, Purves D. Correlated size variations in human visual cortex, lateral geniculate nucleus, and optic tract. J Neurosci. 1997;17:2859–68. 10.1523/JNEUROSCI.17-08-02859.1997. 9092607 10.1523/JNEUROSCI.17-08-02859.1997 PMC6573115

[3] Gong G, He Y, Chen ZJ, Evans AC. Convergence and divergence of thickness correlations with diffusion connections across the human cerebral cortex. NeuroImage. 2012;59:1239–48. 10.1016/J.NEUROIMAGE.2011.08.017. 21884805 10.1016/j.neuroimage.2011.08.017

[4] Yee Y, Fernandes DJ, French L, Ellegood J, Cahill LS, Vousden DA, et al. Structural covariance of brain region volumes is associated with both structural connectivity and transcriptomic similarity. NeuroImage. 2018;179:357–72. 10.1016/J.NEUROIMAGE.2018.05.028. 29782994 10.1016/j.neuroimage.2018.05.028

[5] Romero-Garcia R, Whitaker KJ, Váša F, Seidlitz J, Shinn M, Fonagy P, et al. Structural covariance networks are coupled to expression of genes enriched in supragranular layers of the human cortex. NeuroImage. 2018;171:256. 10.1016/J.NEUROIMAGE.2017.12.060. 29274746 10.1016/j.neuroimage.2017.12.060 PMC5883331

[6] Alexander-Bloch A, Raznahan A, Bullmore E, Giedd J. The convergence of maturational change and structural covariance in human cortical networks. J Neurosci. 2013;33:2889. 10.1523/JNEUROSCI.3554-12.2013. 23407947 10.1523/JNEUROSCI.3554-12.2013 PMC3711653

[7] Raznahan A, Lerch JP, Lee N, Greenstein D, Wallace GL, Stockman M, et al. Patterns of coordinated anatomical change in human cortical development: a longitudinal neuroimaging study of maturational coupling. Neuron. 2011;72:873–84. 10.1016/j.neuron.2011.09.028. 22153381 10.1016/j.neuron.2011.09.028 PMC4870892

[8] Corre C, Friedel M, Vousden DA, Metcalf A, Spring S, Qiu LR, et al. Separate effects of sex hormones and sex chromosomes on brain structure and function revealed by high-resolution magnetic resonance imaging and Spatial navigation assessment of the four core genotype mouse model. Brain Struct Funct. 2016;221:997–1016. 10.1007/s00429-014-0952-0. 25445841 10.1007/s00429-014-0952-0

[9] Forger NG, Rosen GJ, Waters EM, Jacob D, Simerly RB, de Vries GJ. Deletion of Bax eliminates sex differences in the mouse forebrain. Proc Natl Acad Sci U S A. 2004;101:13666–71. 10.1073/pnas.0404644101. 15342910 10.1073/pnas.0404644101 PMC518810

[10] Gorski RA, Gordon JH, Shryne JE, Southam AM. Evidence for a morphological sex difference within the medial preoptic area of the rat brain. Brain Res. 1978;148:333–46. 10.1016/0006-8993(78)90723-0. 656937 10.1016/0006-8993(78)90723-0

[11] Hines M, Allen LS, Gorski RA. Sex differences in subregions of the medial nucleus of the amygdala and the bed nucleus of the stria terminalis of the rat. Brain Res. 1992;579:321–6. 10.1016/0006-8993(92)90068-K. 1352729 10.1016/0006-8993(92)90068-k

[12] Morris JA, Jordan CL, King ZA, Northcutt KV, Breedlove SM. Sexual dimorphism and steroid responsiveness of the posterodorsal medial amygdala in adult mice. Brain Res. 2008;1190:115–21. 10.1016/j.brainres.2007.11.005. 18054901 10.1016/j.brainres.2007.11.005 PMC2258085

[13] Roos J, Roos M, Schaeffer C, Aron C. Sexual differences in the development of accessory olfactory bulbs in the rat. J Comp Neurol. 1988;270:121–31. 10.1002/cne.902700110. 3372734 10.1002/cne.902700110

[14] Segovia S, Orensanz LM, Valencia A, Guillamón A. Effects of sex steroids on the development of the accessory olfactory bulb in the rat: a volumetric study. Brain Res. 1984;16:312–4. 10.1016/0165-3806(84)90036-1. 10.1016/0165-3806(84)90036-1 6498505

[15] Williams RW, Airey DC, Kulkarni A, Zhou G, Lu L. Genetic dissection of the olfactory bulbs of mice: QTLs on four chromosomes modulate bulb size. Behav Genet. 2001;31:1:61–77. 10.1023/A:1010209925783. 11529276 10.1023/a:1010209925783

[16] DeCasien AR, Guma E, Liu S, Raznahan A. Sex differences in the human brain: a roadmap for more careful analysis and interpretation of a biological reality. Biology Sex Differences. 2022;13:43. 10.1186/s13293-022-00448-w. 10.1186/s13293-022-00448-w PMC9327177 35883159

[17] Liu S, Seidlitz J, Blumenthal JD, Clasen LS, Raznahan A. Integrative structural, functional, and transcriptomic analyses of sex-biased brain organization in humans. Proc Natl Acad Sci U S A. 2020;117:18788–98. 10.1073/pnas.1919091117. 32690678 10.1073/pnas.1919091117 PMC7414084

[18] Guma E, Beauchamp A, Liu S, Levitis E, Ellegood J, Pham L, et al. Comparative neuroimaging of sex differences in human and mouse brain anatomy. eLife. 2024;13. 10.7554/eLife.92200.2. 10.7554/eLife.92200 PMC10942785 38488854

[19] Ge R, Liu X, Long D, Frangou S, Vila-Rodriguez F. Sex effects on cortical morphological networks in healthy young adults. NeuroImage. 2021;233:117945. 10.1016/j.neuroimage.2021.117945. 33711482 10.1016/j.neuroimage.2021.117945

[20] Mechelli A, Friston KJ, Frackowiak RS, Price CJ. Structural covariance in the human cortex. J Neurosci. 2005;25:8303. 10.1523/JNEUROSCI.0357-05.2005. 16148238 10.1523/JNEUROSCI.0357-05.2005 PMC6725541

[21] Persson J, Spreng RN, Turner G, Herlitz A, Morell A, Stening E, et al. Sex differences in volume and structural covariance of the anterior and posterior hippocampus. NeuroImage. 2014;99:215–25. 10.1016/j.neuroimage.2014.05.038. 24857714 10.1016/j.neuroimage.2014.05.038

[22] Seitz J, Kubicki M, Jacobs EG, Cherkerzian S, Weiss BK, Papadimitriou G, et al. Impact of sex and reproductive status on memory circuitry structure and function in early midlife using structural covariance analysis. Hum Brain Mapp. 2019;40:1221–33. 10.1002/hbm.24441. 30548738 10.1002/hbm.24441 PMC6365200

[23] Shi Y, Cui D, Niu J, Zhang X, Sun F, Liu H, et al. Sex differences in structural covariance network based on MRI cortical morphometry: effects on episodic memory. Cereb Cortex. 2023;33:8645–53. 10.1093/cercor/bhad147. 37143182 10.1093/cercor/bhad147

[24] Vijayakumar N, Ball G, Seal ML, Mundy L, Whittle S, Silk T. The development of structural covariance networks during the transition from childhood to adolescence. Sci Rep. 2021;11:9451. 10.1038/s41598-021-88918-w. 33947919 10.1038/s41598-021-88918-w PMC8097025

[25] Wierenga LM, Sexton JA, Laake P, Giedd JN, Tamnes CK. A key characteristic of sex differences in the developing brain: greater variability in brain structure of boys than girls. Cereb Cortex. 2018;28:2741–51. 10.1093/cercor/bhx154. 28981610 10.1093/cercor/bhx154 PMC6041809

[26] Wierenga LM, Doucet GE, Dima D, Agartz I, Aghajani M, Akudjedu TN, et al. Greater male than female variability in regional brain structure across the lifespan. Hum Brain Mapp. 2022;43:470–99. 10.1002/hbm.25204. 33044802 10.1002/hbm.25204 PMC8675415

[27] Yang CC, Totzek JF, Lepage M, Lavigne KM. Sex differences in cognition and structural covariance-based morphometric connectivity: evidence from 28,000 + UK biobank participants. Cereb Cortex. 2023;33:10341–54. 10.1093/cercor/bhad286. 37557917 10.1093/cercor/bhad286

[28] Cahill LS, Laliberté CL, Ellegood J, Spring S, Gleave JA, van Eede MC, et al. Preparation of fixed mouse brains for MRI. NeuroImage. 2012;60:933–9. 10.1016/j.neuroimage.2012.01.100. 22305951 10.1016/j.neuroimage.2012.01.100

[29] Lerch JP, Sled JG, Henkelman RM. MRI Phenotyping of Genetically Altered Mice. Methods in Molecular Biology. 2011:349– 61; 10.1007/978-1-61737-992-5_17 10.1007/978-1-61737-992-5_17 21279611

[30] Spring S, Lerch JP, Henkelman RM. Sexual dimorphism revealed in the structure of the mouse brain using three-dimensional magnetic resonance imaging. NeuroImage. 2007;35:1424–33. 10.1016/j.neuroimage.2007.02.023. 17408971 10.1016/j.neuroimage.2007.02.023

[31] Ellegood J, Anagnostou E, Babineau BA, Crawley JN, Lin L, Genestine M, et al. Clustering autism: using neuroanatomical differences in 26 mouse models to gain insight into the heterogeneity. Mol Psychiatry. 2015;20:118–25. 10.1038/mp.2014.98. 25199916 10.1038/mp.2014.98 PMC4426202

[32] Spencer Noakes TL, Henkelman RM, Nieman BJ. Partitioning k-space for cylindrical three‐dimensional rapid acquisition with relaxation enhancement imaging in the mouse brain. NMR Biomed. 2017;30. 10.1002/nbm.3802. 10.1002/nbm.3802 28902423

[33] Avants B, Tustison NJ, Song G. Advanced normalization tools: V1.0. Insight J. 2009. 10.54294/uvnhin.

[34] Avants BB, Tustison NJ, Song G, Cook PA, Klein A, Gee JC. A reproducible evaluation of ants similarity metric performance in brain image registration. NeuroImage. 2011;54:2033–44. 10.1016/j.neuroimage.2010.09.025. 20851191 10.1016/j.neuroimage.2010.09.025 PMC3065962

[35] Collins DL, Neelin P, Peters TM, Evans AC. Automatic 3D intersubject registration of MR volumetric data in standardized Talairach space. J Comput Assist Tomogr. 1994;18:192–205. 8126267

[36] Eskildsen SF, Coupé P, Fonov V, Manjón JV, Leung KK, Guizard N, et al. BEaST: brain extraction based on nonlocal segmentation technique. NeuroImage. 2012;59:2362–73. 10.1016/j.neuroimage.2011.09.012. 21945694 10.1016/j.neuroimage.2011.09.012

[37] Friedel M, van Eede MC, Pipitone J, Chakravarty MM, Lerch JP. Pydpiper: a flexible toolkit for constructing novel registration pipelines. Front Neuroinformatics. 2014;8. 10.3389/fninf.2014.00067. 10.3389/fninf.2014.00067 PMC4115634 25126069

[38] Chung MK, Worsley KJ, Paus T, Cherif C, Collins DL, Giedd JN, et al. A unified statistical approach to Deformation-Based morphometry. NeuroImage. 2001;14:595–606. 10.1006/nimg.2001.0862. 11506533 10.1006/nimg.2001.0862

[39] Chakravarty MM, Steadman P, van Eede MC, Calcott RD, Gu V, Shaw P, et al. Performing label-fusion-based segmentation using multiple automatically generated templates. Hum Brain Mapp. 2013;34:2635–54. 10.1002/hbm.22092. 22611030 10.1002/hbm.22092 PMC4896505

[40] Pipitone J, Park MTM, Winterburn J, Lett TA, Lerch JP, Pruessner JC, et al. Multi-atlas segmentation of the whole hippocampus and subfields using multiple automatically generated templates. NeuroImage. 2014;101:494–512. 10.1016/j.neuroimage.2014.04.054. 24784800 10.1016/j.neuroimage.2014.04.054

[41] Dorr AE, Lerch JP, Spring S, Kabani N, Henkelman RM. High resolution three-dimensional brain atlas using an average magnetic resonance image of 40 adult C57Bl/6J mice. NeuroImage. 2008;42:60–9. 10.1016/j.neuroimage.2008.03.037. 18502665 10.1016/j.neuroimage.2008.03.037

[42] Qiu LR, Fernandes DJ, Szulc-Lerch KU, Dazai J, Nieman BJ, Turnbull DH, et al. Mouse MRI shows brain areas relatively larger in males emerge before those larger in females. Nat Commun. 2018;9:2615. 10.1038/s41467-018-04921-2. 29976930 10.1038/s41467-018-04921-2 PMC6033927

[43] Richards K, Watson C, Buckley RF, Kurniawan ND, Yang Z, Keller MD, et al. Segmentation of the mouse hippocampal formation in magnetic resonance images. NeuroImage. 2011;58:732–40. 10.1016/j.neuroimage.2011.06.025. 21704710 10.1016/j.neuroimage.2011.06.025

[44] Steadman PE, Ellegood J, Szulc KU, Turnbull DH, Joyner AL, Henkelman RM, et al. Genetic effects on cerebellar structure across mouse models of autism using a magnetic resonance imaging atlas. Autism Res. 2014;7:124–37. 10.1002/aur.1344. 24151012 10.1002/aur.1344 PMC4418792

[45] Ullmann JFP, Watson C, Janke AL, Kurniawan ND, Reutens DC. A segmentation protocol and MRI atlas of the C57BL/6J mouse neocortex. NeuroImage. 2013;78:196–203. 10.1016/j.neuroimage.2013.04.008. 23587687 10.1016/j.neuroimage.2013.04.008

[46] Fortin J-P, Parker D, Tunç B, Watanabe T, Elliott MA, Ruparel K, et al. Harmonization of multi-site diffusion tensor imaging data. NeuroImage. 2017;161:149–70. 10.1016/j.neuroimage.2017.08.047. 28826946 10.1016/j.neuroimage.2017.08.047 PMC5736019

[47] Fortin J-P, Cullen N, Sheline YI, Taylor WD, Aselcioglu I, Cook PA, et al. Harmonization of cortical thickness measurements across scanners and sites. NeuroImage. 2018;167:104–20. 10.1016/j.neuroimage.2017.11.024. 29155184 10.1016/j.neuroimage.2017.11.024 PMC5845848

[48] Johnson WE, Li C, Rabinovic A. Adjusting batch effects in microarray expression data using empirical Bayes methods. Biostatistics. 2007;8:118–27. 10.1093/biostatistics/kxj037. 16632515 10.1093/biostatistics/kxj037

[49] Leek JT, Johnson WE, Parker HS, Fertig EJ, Jaffe AE, Zhang Y, et al. Sva: surrogate variable analysis. Bioinformatics. 2022;17(28):882–3. 10.1093/bioinformatics/bts034.

[50] Van Essen DC, Ugurbil K, Auerbach E, Barch D, Behrens TEJ, Bucholz R, et al. The human connectome project: A data acquisition perspective. NeuroImage. 2012;62:2222–31. 10.1016/j.neuroimage.2012.02.018. 22366334 10.1016/j.neuroimage.2012.02.018 PMC3606888

[51] Glasser MF, Sotiropoulos SN, Wilson JA, Coalson TS, Fischl B, Andersson JL, et al. The minimal preprocessing pipelines for the human connectome project. NeuroImage. 2013;80:105–24. 10.1016/j.neuroimage.2013.04.127. 23668970 10.1016/j.neuroimage.2013.04.127 PMC3720813

[52] Dale A, Fischl B, Sereno MI. Cortical surface-Based analysis: I. Segmentation and surface reconstruction. NeuroImage. 1999;9:179–94. 9931268 10.1006/nimg.1998.0395

[53] Rosen AFG, Roalf DR, Ruparel K, Blake J, Seelaus K, Villa LP, et al. Quantitative assessment of structural image quality. NeuroImage. 2018;169:407–18. 10.1016/j.neuroimage.2017.12.059. 29278774 10.1016/j.neuroimage.2017.12.059 PMC5856621

[54] Fischl B, FreeSurfer. NeuroImage. 2012;62:774–81. 10.1016/j.neuroimage.2012.01.021. 22248573 10.1016/j.neuroimage.2012.01.021 PMC3685476

[55] Dale AM, Sereno MI. Improved localizadon of cortical activity by combining EEG and MEG with MRI cortical surface reconstruction: A linear approach. J Cogn Neurosci. 1993;5:162–76. 10.1162/jocn.1993.5.2.162. 23972151 10.1162/jocn.1993.5.2.162

[56] Fischl B, Dale AM, Sereno MI, Tootell RBH, Rosen BR. A coordinate system for the cortical surface. NeuroImage. 1998;7:S740. 10.1016/S1053-8119(18)31573-8.

[57] Fischl B, Sereno MI, Tootell RBH, Dale AM. High-resolution intersubject averaging and a coordinate system for the cortical surface. Hum Brain Mapp. 1999;8:272–84. 10.1002/(SICI)1097-0193(1999)8:4-272::AID-HBM10-3.0.CO;2-4. 10619420 10.1002/(SICI)1097-0193(1999)8:4<272::AID-HBM10>3.0.CO;2-4 PMC6873338

[58] Fischl B, Sereno MI, Dale A. Cortical Surface-Based analysis: II: inflation, flattening, and a Surface-Based coordinate system. NeuroImage. 1999;9:195–207. 9931269 10.1006/nimg.1998.0396

[59] Fischl B, Dale AM. Measuring the thickness of the human cerebral cortex from magnetic resonance images. Proc Natl Acad Sci U S A. 2000;97:11050–5. 10984517 10.1073/pnas.200033797 PMC27146

[60] Fischl B, Liu A, Dale AM. Automated manifold surgery: constructing geometrically accurate and topologically correct models of the human cerebral cortex. IEEE Trans Med Imaging. 2001;20:70–80. 11293693 10.1109/42.906426

[61] Fischl B, Salat DH, Busa E, Albert M, Dieterich M, Haselgrove C, et al. Whole brain segmentation: automated labeling of neuroanatomical structures in the human brain. Neuron. 2002;33:341–55. 11832223 10.1016/s0896-6273(02)00569-x

[62] Kuperberg GR, Broome M, McGuire PK, David AS, Eddy M, Ozawa F, et al. Regionally localized thinning of the cerebral cortex in schizophrenia. Arch Gen Psychiatry. 2003;60:878–88. 12963669 10.1001/archpsyc.60.9.878

[63] Desikan RS, Ségonne F, Fischl B, Quinn BT, Dickerson BC, Blacker D, et al. An automated labeling system for subdividing the human cerebral cortex on MRI scans into gyral based regions of interest. NeuroImage. 2006;31:968–80. 10.1016/j.neuroimage.2006.01.021. 16530430 10.1016/j.neuroimage.2006.01.021

[64] Jovicich J, Czanner S, Greve D, Haley E, van der Kouwe A, Gollub R, et al. Reliability in multi-site structural MRI studies: effects of gradient non-linearity correction on Phantom and human data. NeuroImage. 2006;30:436–43. 10.1016/j.neuroimage.2005.09.046. 16300968 10.1016/j.neuroimage.2005.09.046

[65] Han X, Jovicich J, Salat D, van der Kouwe A, Quinn B, Czanner S, et al. Reliability of MRI-derived measurements of human cerebral cortical thickness: the effects of field strength, scanner upgrade and manufacturer. NeuroImage. 2006;32:180–94. 16651008 10.1016/j.neuroimage.2006.02.051

[66] Reuter M, Rosas HD, Fischl B. Highly accurate inverse consistent registration: A robust approach. NeuroImage. 2010;53:1181–96. 10.1016/j.neuroimage.2010.07.020. 20637289 10.1016/j.neuroimage.2010.07.020 PMC2946852

[67] Zaretskaya N, Fischl B, Reuter M, Renvall V, Polimeni JR. Advantages of cortical surface reconstruction using submillimeter 7 T MEMPRAGE. NeuroImage. 2018;165:11–26. 10.1016/j.neuroimage.2017.09.060. 28970143 10.1016/j.neuroimage.2017.09.060 PMC6383677

[68] Fischl B, Salat DH, van der Kouwe AJW, Makris N, Ségonne F, Quinn BT, et al. Sequence-independent segmentation of magnetic resonance images. NeuroImage. 2004;23:S69 - S84; doi:DOI: 10.1016/j.neuroimage.2004.07.016. 10.1016/j.neuroimage.2004.07.016 15501102

[69] Glasser MF, Coalson TS, Robinson EC, Hacker CD, Harwell J, Yacoub E, et al. A multi-modal parcellation of human cerebral cortex. Nature. 2016;536:171–8. 10.1038/nature18933. 27437579 10.1038/nature18933 PMC4990127

[70] Fischl B, van der Kouwe A, Destrieux C, Halgren E, Ségonne F, Salat DH, et al. Automatically parcellating the human cerebral cortex. Cereb Cortex. 2004;14:11–22. 10.1093/cercor/bhg087. 14654453 10.1093/cercor/bhg087

[71] Iglesias JE, Augustinack JC, Nguyen K, Player CM, Player A, Wright M, et al. A computational atlas of the hippocampal formation using ex vivo, ultra-high resolution MRI: application to adaptive segmentation of in vivo MRI. NeuroImage. 2015;115:117–37. 10.1016/j.neuroimage.2015.04.042. 25936807 10.1016/j.neuroimage.2015.04.042 PMC4461537

[72] Iglesias JE, Van Leemput K, Bhatt P, Casillas C, Dutt S, Schuff N, et al. Bayesian segmentation of brainstem structures in MRI. NeuroImage. 2015;113:184–95. 10.1016/j.neuroimage.2015.02.065. 25776214 10.1016/j.neuroimage.2015.02.065 PMC4434226

[73] Saygin ZM, Kliemann D, Iglesias JE, van der Kouwe AJW, Boyd E, Reuter M, et al. High-resolution magnetic resonance imaging reveals nuclei of the human amygdala: manual segmentation to automatic atlas. NeuroImage. 2017;155:370–82. 10.1016/j.neuroimage.2017.04.046. 28479476 10.1016/j.neuroimage.2017.04.046 PMC5557007

[74] Neudorfer C, Germann J, Elias GJB, Gramer R, Boutet A, Lozano AM. A high-resolution in vivo magnetic resonance imaging atlas of the human hypothalamic region. Sci Data. 2020;7:305. 10.1038/s41597-020-00644-6. 32934244 10.1038/s41597-020-00644-6 PMC7492465

[75] Devenyi G. Library of Bpipe functions for processing Minc files, version c7561d6. 2024.

[76] Benjamini Y, Hochberg Y. Controlling the false discovery rate: A practical and powerful approach to multiple testing. J Roy Stat Soc: Ser B (Methodol). 1995;57:289–300. 10.1111/j.2517-6161.1995.tb02031.x.

[77] Benjamini Y, Yekutieli D. The control of the false discovery rate in multiple testing under dependency. Ann Stat. 2001;29. 10.1214/aos/1013699998.

[78] cocoframer. © 2018 Allen Institute for Brain Science. Allen Brain Explorer. Available from: connectivity.brain-map.org/3d-viewer/. Allen Institute for Brain Science2018.

[79] Glur C. data.tree: General Purpose Hierarchical Data Structure. 2020.

[80] Kuhn M, Wickham H. Tidymodels: a collection of packages for modeling and machine learning using tidyverse principles. 2020.

[81] Landau WM. The targets R package: a dynamic Make-like function-oriented pipeline toolkit for reproducibility and high-performance computing. J Open Source Softw. 2021;6:2959. 10.21105/joss.02959.

[82] Lander JP. useful: A Collection of Handy, Useful Functions. 2018.

[83] Lerch J, MRIcrotome. Visualization Tools for 3D Volumes. 2023.

[84] Lerch J, Hammill C, van Eede M, Cassel D. RMINC: Statistical Tools for Medical Imaging NetCDF (MINC) Files. 2017.

[85] Mowinckel AM, Vidal-Piñeiro D. Visualisation of Brain Statistics with R-packages ggseg and ggseg3d. 2019.

[86] Mowinckel AM, Vidal-Piñeiro D, ggsegGlasser. Glasser datasets for the ggseg-plotting tool. 2023.

[87] Mowinckel AM. Vidal-Piñeiro D. ggseg: Plotting Tool for Brain Atlases. 2022.

[88] Pedersen TL. patchwork: The Composer of Plots. 2024.

[89] Robinson D, Hayes A, Couch S, editors. broom: Convert Statistical Objects into Tidy Tibbles. 2023.

[90] Wickham H, Averick M, Bryan J, Chang W, McGowan LDA, François R, et al. Welcome to the tidyverse. J Open Source Softw. 2019;4:1686. 10.21105/joss.01686.

[91] Wickham H. reshape2: Flexibly Reshape Data: A Reboot of the Reshape Package. 2020.

[92] Hallgrímsson B, Maiorana V. Variability and size in mammals and birds. Biol J Linn Soc. 2000;70:571–95. 10.1111/j.1095-8312.2000.tb00218.x.

[93] He Y, Chen ZJ, Evans AC. Small-World anatomical networks in the human brain revealed by cortical thickness from MRI. Cereb Cortex. 2007;17:2407–19. 10.1093/cercor/bhl149. 17204824 10.1093/cercor/bhl149

[94] DuPre E, Spreng RN. Structural covariance networks across the life span, from 6 to 94 years of age. Netw Neurosci. 2017;1:302–23. 10.1162/NETN_a_00016. 29855624 10.1162/NETN_a_00016 PMC5874135

[95] DeCasien AR, Tsai K, Liu S, Thomas A, Raznahan A. Evolutionary divergence between homologous X-Y chromosome genes shapes sex-biased biology. Nat Ecol Evol. 2024. 10.1038/s41559-024-02627-x. 10.1038/s41559-024-02627-x PMC13051466 39856216

[96] Oh SW, Harris JA, Ng L, Winslow B, Cain N, Mihalas S, et al. A mesoscale connectome of the mouse brain. Nature. 2014;508:7495207–14. 10.1038/nature13186. 10.1038/nature13186 PMC5102064 24695228

[97] Harris JA, Mihalas S, Hirokawa KE, Whitesell JD, Choi H, Bernard A, et al. Hierarchical organization of cortical and thalamic connectivity. Nature. 2019;575:7781:195–202. 10.1038/s41586-019-1716-z. 31666704 10.1038/s41586-019-1716-z PMC8433044

[98] Ghaziri J, Tucholka A, Girard G, Houde J-C, Boucher O, Gilbert G, et al. The corticocortical structural connectivity of the human Insula. Cereb Cortex. 2015;27(2):1216–28. 10.1093/cercor/bhv308. 10.1093/cercor/bhv308 26683170

[99] Allen Institute for Brain Science. Experiment 177889243. Allen Institute for Brain Science. http://connectivity.brain-map.org/projection/experiment/177889243

[100] Neaux D, Blanc B, Ortiz K, Locatelli Y, Schafberg R, Herrel A, et al. Constraints associated with captivity alter craniomandibular integration in wild Boar. J Anat. 2021;239:2489–97. 10.1111/joa.13425. 10.1111/joa.13425 PMC8273579 33713426

[101] Zajitschek SRK, Zajitschek F, Bonduriansky R, Brooks RC, Cornwell W, Falster DS, et al. Sexual dimorphism in trait variability and its eco-evolutionary and statistical implications. eLife. 2020;9:e63170. 10.7554/eLife.63170. 33198888 10.7554/eLife.63170 PMC7704105

[102] Bölte S, Neufeld J, Marschik PB, Williams ZJ, Gallagher L, Lai M-C. Sex and gender in neurodevelopmental conditions. Nat Reviews Neurol. 2023;19:3. 10.1038/s41582-023-00774-6. 10.1038/s41582-023-00774-6 PMC10154737 36747038

[103] Nadig A, Seidlitz J, McDermott CL, Liu S, Bethlehem R, Moore TM et al. Morphological integration of the human brain across adolescence and adulthood. Proceedings of the National Academy of Sciences. 2021;118:14:e2023860118; 10.1073/pnas.2023860118 10.1073/pnas.2023860118 PMC8040585 33811142

[104] Beauchamp A, Yee Y, Darwin BC, Raznahan A, Mars RB, Lerch JP. Whole-brain comparison of rodent and human brains using Spatial transcriptomics. eLife. 2022;11:e79418. 10.7554/eLife.79418. 36342372 10.7554/eLife.79418 PMC9708081

[105] Arnold AP, Chen X. What does the four core genotypes mouse model tell Us about sex differences in the brain and other tissues? Front Neuroendocrinol. 2009;30:1–9. 10.1016/j.yfrne.2008.11.001. 19028515 10.1016/j.yfrne.2008.11.001 PMC3282561

[106] Barron HC, Mars RB, Dupret D, Lerch JP, Sampaio-Baptista C. Cross-species neuroscience: closing the explanatory gap. Philosophical Trans Royal Soc B. 2021;376:20190633. 10.1098/rstb.2019.0633. 10.1098/rstb.2019.0633 PMC7116399 33190601

